# Direct-Acting Antivirals in Patients with Comorbidities for the Simplified Management of HCV Infection: An Expert Review with a Focus on Sofosbuvir–Velpatasvir

**DOI:** 10.3390/v18080854

**Published:** 2026-08-04

**Authors:** Alessio Aghemo, Alessia Ciancio, Ernesto Claar, Nicola Coppola, Alessandra Mangia, Marco Riglietta, Massimo Puoti

**Affiliations:** 1 Department of Biomedical Sciences, Humanitas University, 20072 Pieve Emanuele, Italy; alessio.aghemo@hunimed.eu; 2 Division of Internal Medicine and Hepatology, IRCCS Humanitas Research Hospital, 20089 Rozzano, Italy; 3 A.O.U. Città della Salute e della Scienza di Torino, 10126 Torino, Italy; alessia.ciancio@unito.it; 4 Ospedale Evangelico Villa Betania, 80147 Napoli, Italy; ernestoclaar@gmail.com; 5 Dipartimento di Salute Mentale e Fisica e Medicina Preventiva, Università degli Studi della Campania “Luigi Vanvitelli”, 80133 Napoli, Italy; nicola.coppola@unicampania.it; 6 IRCCS Casa Sollievo della Sofferenza, 71013 San Giovanni Rotondo, Italy; a.mangia@operapadrepio.it; 7 ASST Papa Giovanni XXIII, 24127 Bergamo, Italy; mriglietta@asst-pg23.it; 8 ASST Grande Ospedale Metropolitano Niguarda-Università degli Studi di Milano Bicocca, 20162 Milano, Italy

**Keywords:** HCV infection, comorbidities, polypharmacy, direct-acting antivirals, drug–drug interactions, sofosbuvir/velpatasvir

## Abstract

Introduction: Hepatitis C virus (HCV) infection often coexists with comorbidities, increasing vulnerability, complications, and adverse events. Direct-acting antivirals (DAAs) have dramatically improved HCV management, but they differ in drug–drug interaction (DDI) profiles. Sofosbuvir/velpatasvir (SOF/VEL) is associated with minimal clinically relevant interactions. Areas covered: A narrative review of the literature was conducted by searching PubMed and major international guidelines, focusing on studies published in the DAA era addressing HCV patients with major comorbidities, focusing on diabetes, metabolic syndrome, and cardiovascular disease; neuropsychiatric disorders; cancer; transplants; and use of substances or treatment with opioid agonists; and patients requiring hormone therapy including transgender patients. Expert opinion: Based on the literature and real-world data, managing polypharmacy in HCV patients with comorbidities is effective and well-tolerated, provided thorough drug review, potential DDI analysis, proactive monitoring, and coordinated multidisciplinary care are ensured. DAAs have dramatically improved the management of HCV patients; however, they have different DDI profiles that should be carefully checked. SOF/VEL has been shown to be associated with minimal clinically relevant interactions and offers a simple dosing regimen. DAA treatment is strongly advised in HCV comorbid patients not only to cure HCV but also to improve the course of comorbidities, provided that DDIs are no longer considered mere minor details.

## 1. Introduction

Hepatitis C virus (HCV) infection represents a highly relevant clinical challenge, particularly due to its frequent association with comorbidities that complicate patient management. Numerous studies highlight how conditions such as diabetes, renal insufficiency, cardiovascular diseases, and co-infections contribute to making these patients particularly vulnerable and at increased risk of adverse events. In this context, the need for therapeutic regimens that are simple, are safe, and have a low pharmacological impact becomes essential for optimizing adherence and minimizing drug interactions [1].

Polypharmacy remains a central concern in patients with multiple comorbidities, as the risk of drug–drug interactions (DDIs) can compromise both the efficacy and safety of antiviral therapy. Sofosbuvir/velpatasvir (SOF/VEL) has few and limited clinically significant DDIs and a straightforward dosing schedule [2]. This operational advantage is particularly relevant in integrated care pathways, where multidisciplinary teams must coordinate the management of HCV alongside comorbid psychiatric, metabolic, and renal conditions. By prioritizing regimens with lower pharmacological impact, clinicians can optimize patient outcomes while maintaining a high standard of safety.

The SOF/VEL combination has demonstrated pangenotypic efficacy and tolerability in diverse patient populations, including those with multiple comorbidities or complex clinical presentations. Evidence from recent studies indicates consistently high rates of sustained virologic response (SVR) and a favorable safety profile, even among individuals with advanced disease or additional health burdens [3,4].

This Expert Opinion Paper aims to critically evaluate the efficacy, safety, and practical considerations of SOF/VEL in the context of chronic renal insufficiency, diabetes mellitus, and psychiatric conditions; in patients undergoing hormonal treatments, including transgender individuals; and in people who use drugs (PWUD) or participate in addiction treatment programs. A particular emphasis is placed on the challenges posed by polypharmacy, including DDIs.

The objective is to provide a balanced and current review that can support the simplified and integrated management of HCV in those with multifaceted comorbidities. The recommendations presented herein are intended to assist clinicians in adopting evidence-based approaches that streamline care and address the unique challenges faced by these complex patient groups, ultimately aiming to improve clinical outcomes and support SVR.

## 2. Patients with Chronic Kidney Disease

Chronic kidney disease (CKD) is highly prevalent among patients with HCV infection, and this can be attributed to a combination of clinical and demographic factors [5]. First, the advanced age commonly observed in certain countries in the HCV patient population naturally increases the risk of CKD, as renal function tends to decline with age. Additionally, these patients are often subject to polypharmacy due to multiple coexisting health conditions, which can further strain renal function and complicate management. On the other hand, effective HCV treatment leading to SVR lowers the likelihood of developing CKD, improves extra-hepatic outcomes, and is associated with enhanced overall survival [6,7]. Currently all pangenotypic antiviral agents can now be used both safely and effectively in patients with CKD. Routine renal function assessment is no longer a limiting factor for regimen selection, given the safety of current pangenotypic DAAs across CKD stages [1,7].

Historically, the use of sofosbuvir-based antiviral regimens in patients with severely reduced renal function (estimated glomerular filtration rate [eGFR] < 30 mL/min) was approached with caution. This was due to concerns regarding the potential accumulation of sofosbuvir metabolites, which are primarily cleared renally, and the theoretical risk of toxicity. However, subsequent clinical studies have robustly challenged these initial reservations. Accumulating evidence now demonstrates that sofosbuvir-containing regimens are both safe and effective in patients with advanced CKD, including those on dialysis [8,9,10,11,12]. These findings have significantly broadened treatment options and improved outcomes for this vulnerable population. In particular, the SOF/VEL combination was shown to be safe and effective in HCV patients with CKD [13,14,15,16,17]. A systematic review and meta-analysis assessing SOF/VEL for the treatment of chronic hepatitis C (CHC) in patients with end-stage renal disease (ESRD) on renal replacement therapy found a pooled estimate of efficacy of SOF/VEL among patients with cirrhosis of nearly 92%. No serious adverse events attributable to SOF and VEL were reported in the included studies. In a real-life prospective study in patients with CHC in ESRD on maintenance hemodialysis, all 51 patients achieved SVR at 12 weeks (SVR12) with none of the patients reporting any serious adverse events [14]. The performance of SOF/VEL with or without low-dose ribavirin in HCV-infected patients with CKD stage 4 or 5 was evaluated in 191 patients. The SVR12 rates were above 90% in patients with both compensated and decompensated liver disease and the treatment was well-tolerated [15]. A prospective cohort study in 220 patients treated with SOF/VEL for CHC in ESRD and undergoing hemodialysis showed improvements in renal function, including a reduction in the frequency of weekly dialysis sessions and an SVR rate of 91.4%. A single-tablet SOF/VEL regimen administered over 12 weeks demonstrated safety, efficacy, and good tolerability, irrespective of the underlying cause of ESRD, the presence of cirrhosis-related complications, the HCV genotype, or prior treatment history. Achieving SVR through successful HCV treatment reduces the risk of ESRD-related complications, improves extra-hepatic symptoms, and significantly improves survival outcomes [16].

## 3. Patients with Diabetes, Metabolic Syndrome, and Cardiovascular Disease

### 3.1. Diabetes

The association between chronic HCV infection and glucose metabolism disease (insulin resistance and type 2 diabetes mellitus) has been known for a long time and is supported by epidemiological evidence and meta-analyses. Insulin resistance (IR) normally precedes type 2 diabetes mellitus (T2DM) and is strongly associated with its development. In Italy, a very recent real-world study examined individuals aged ≥55 years with HCV infection, comparing those with and without a co-diagnosis of T2DM. Findings revealed that the coexistence of T2DM in HCV+ patients was associated with poorer health status, increased hospitalization needs, and more complex polypharmacy, highlighting the importance of tailored management in this population [18].

In the era of DAAs, SVR has been repeatedly associated with improved glycemic control in patients with diabetes, documented by reductions in HbA1c and/or fasting plasma glucose in prospective–retrospective studies and real-world cohorts [19,20]. A recent meta-analysis found a significant reduction in HbA1c after SVR in patients with T2DM, suggesting a measurable metabolic benefit in the short-to-intermediate term following DAA therapy [21]. Consistently, studies in patients with and without cirrhosis suggest that liver disease severity may modulate the trajectory of glucose metabolism after HCV eradication, supporting the need for continued clinical surveillance even after SVR [22]. However, the magnitude and especially the durability of the effect are not uniform, and longitudinal studies indicate that HbA1c reduction may be pronounced in the early post-treatment phase but tends to attenuate during longer follow-up, eventually converging towards trends similar to those observed in untreated patients or non-responders [23,24]. This potential “transience” supports the hypothesis that viral clearance removes the HCV-related component of IR, while other determinants (obesity, sedentary lifestyle, diet, diabetes pharmacotherapy, and comorbidities) continue to drive long-term metabolic risk [25]. Certain clinical factors appear to be associated with a higher likelihood of glycemic improvement (e.g., shorter diabetes duration and less advanced liver disease), whereas conditions such as cirrhosis and unfavorable BMI may blunt or negate the short-term metabolic advantage [24]. Even in non-diabetic individuals, DAA therapy has been associated with improvements in IR parameters (e.g., Homeostatic Model Assessment of Insulin Resistance [HOMA]-IR) during follow-up, suggesting a metabolic effect that may precede or help prevent glycemic deterioration in at-risk populations [26]. From a safety perspective, an increased risk of “reported” hypoglycemia has been described when DAAs are co-prescribed with antidiabetic medications, particularly insulin and sulfonylureas, which is more evident in frail patients (advanced age and/or advanced liver disease) [27]. In the same pharmacovigilance analysis, co-prescription of DAAs with biguanides, DPP-4 inhibitors, or GLP-1 receptor agonists (GLP-1 RAs) was not associated with a significant increase in hypoglycemia reports, suggesting a more favorable profile compared with classes of antidiabetics carrying higher hypoglycemic risk [27]. Pathophysiological data consistent with a post-eradication metabolic “reset” include the observation of increased endogenous serum GLP-1 levels after SVR in a DAA-treated cohort, indicating that viral clearance may also influence hormonal axes related to appetite and metabolism [28]. Accordingly, during the first weeks of DAA treatment and in the immediate post-treatment period, it is prudent to intensify glucose self-monitoring and to promptly reassess antidiabetic dosing/strategies in patients at risk for hypoglycemia; furthermore, maintaining long-term follow-up may prevent the misleading perception of “resolved diabetes” after SVR. Based on the hypothesis that chronic HCV infection contributes to IR and that its elimination may improve glucose metabolism, a Taiwan investigation, drawing on data from the National HCV Registry, evaluated whether HCV eradication by SOF/VEL has an impact on glycemic control, as measured by HbA1c, in patients with CHC and T2DM or a prediabetic state [29]. Researchers analyzed 2180 eligible patients, of whom 695 had T2DM. In the diabetic cohort, mean HbA1c levels dropped significantly from 7.32 ± 1.72% at baseline to 6.87 ± 1.34% following achievement of SVR12. Notably, those with higher initial HbA1c values and cirrhosis experienced more substantial reductions in HbA1c. Among diabetic patients whose HbA1c was ≥6.5% before treatment, 24.6% reached levels below 6.5% after HCV clearance, while 24.4% of individuals with prediabetes saw their HbA1c fall below 5.7% [29]. These reductions were both statistically and clinically significant, suggesting improved glycemic control after HCV clearance. The subgroups with the greatest reduction in HbA1c were patients with higher baseline HbA1c and those with cirrhosis. The clinical interpretation is that the effect of HCV eradication is more evident in those with greater metabolic or hepatic decompensation. Treatment with SOF/VEL was generally well-tolerated. These results indicate that successful HCV therapy can enhance glycemic control, underscoring the importance of coordinated care between hepatology and other medical specialists.

### 3.2. Metabolic Syndrome and Cardiovascular Risk

Patients with HCV infection and metabolic syndrome (visceral obesity, dyslipidemia, hypertension, impaired glucose tolerance/diabetes) represent an even greater clinical challenge, considering that cardiovascular (CV) risk often also competes with the risk associated with fibrosis progression. HCV infection has been associated with an increased incidence of CV events and mortality (particularly in populations with a high prevalence of diabetes and hypertension), likely mediated by chronic inflammation, endothelial dysfunction, and worsening insulin resistance [30]. In this setting, antiviral therapy should be integrated with structured management of cardiometabolic risk factors and polypharmacy in line with international recommendations [31].

Beyond hepatic and metabolic benefits, HCV eradication with DAAs also appears to translate into improved CV outcomes in real-world cohorts, including a reduction in major adverse CV events among individuals with prediabetes and active infection, and better CV survival among patients achieving SVR, although diabetes and CKD remain adverse prognostic factors [32,33]. From a metabolic standpoint, after SVR, it is common to observe changes (sometimes an “apparent worsening”) in the lipid profile, with increases in total cholesterol and LDL cholesterol [34,35]. This is not necessarily an iatrogenic effect but may reflect the unmasking of virus-related hypolipidemia and/or shifts in lipid metabolism after viral clearance [36]. A post-treatment lipid panel should therefore be obtained (e.g., at SVR12), and statin/lipid-lowering therapy should be adjusted according to overall CV risk, bearing in mind that certain DAA–statin combinations require temporary discontinuation or dose reduction during antiviral treatment [37].

### 3.3. Atrial Fibrillation

Atrial fibrillation (AF) is common in the metabolically complex patient and may coexist with advanced liver disease, where the balance between thrombosis and bleeding becomes more delicate (thrombocytopenia, varices, and liver disease with a “rebalanced” hemostatic state). A key practical issue is whether anticoagulation, especially with direct oral anticoagulants (DOACs), can be continued during DAA therapy. Pharmacological concerns include modulation of P-glycoprotein and CYP3A4/transporters, potentially increasing DOAC exposure and bleeding risk, particularly with specific drug classes or regimens [38]. Reviews focused on interactions between antithrombotic agents and DAAs provide a clear rationale and a practical risk map [39]. Real-world multicenter data suggest that DAA–DOAC coadministration is often feasible and overall safe with appropriate selection and monitoring: McDaniel et al. [40] support “safe coadministration,” and the Italian analysis by Rosato et al. [38] on sofosbuvir-based DAAs is consistent with this approach. In practice, a reasonable approach is to: (1) choose, when possible, a DAA regimen with lower interaction potential with the DOAC in use; (2) assess renal and hepatic function, age, bleeding history, and concomitant therapies (antiplatelet drugs, NSAIDs); (3) plan closer clinical monitoring (bleeding symptoms, complete blood count) during the 8–12 weeks of antiviral therapy. Finally, the potential interaction between sofosbuvir-containing regimens and amiodarone requires particular attention: there have been reports of severe bradycardia and asystole with sofosbuvir (also across different combinations) [41]. Therefore, in patients receiving amiodarone, management decisions should be shared with a cardiologist, considering substitution when possible, and/or strict monitoring.

Clinically relevant DAA interactions with antidiabetic and cardiovascular drugs are summarized in Table 1.

## 4. Patients with Neuropsychiatric Disorders

HCV is well recognized for its hepatic tropism, but increasing evidence supports the virus’s ability to infect and replicate within the central nervous system (CNS). Molecular biology studies have identified viral RNA and HCV proteins in glial cells and neurons, suggesting direct neuroinvasion and possible viral persistence in the CNS. Patients with chronic hepatitis often exhibit a high prevalence of psychiatric disorders, including cognitive impairment, mood disturbances, and anxiety, which are further complicated by frequent substance use and psychiatric comorbidities. The chronicity of HCV infection can significantly affect mental health, leading to elevated levels of stress, depression, and anxiety [42]. Moreover, individuals living with HCV frequently experience social marginalization, stigma, and increased vulnerability, all of which can exacerbate the psychological burden and complicate disease management. Understanding these interconnected issues is essential for providing comprehensive care to this complex patient population.

### 4.1. Neuropsychiatric Profile of HCV Patients

Major depressive disorder is notably prevalent among patients with chronic HCV infection. Studies have shown that up to 30–50% of individuals with chronic HCV may experience clinically significant depressive symptoms. This elevated risk is attributed to both direct viral effects on the CNS and inflammatory cytokine release and psychosocial factors like stigma and chronic illness burden [43]. Anxiety disorders, including generalized anxiety disorder, panic disorder, and adjustment disorders, are also frequently observed in HCV-infected populations.

### 4.2. Antiviral Therapy in Populations with Psychiatric Disorders

New DAAs are much safer than older interferon-based therapies, which frequently caused or worsened psychiatric symptoms. The introduction of DAAs has really revolutionized the management of HCV infection. There is wide evidence showing that DAAs offer a highly effective and safe treatment option that enhances quality of life for patients with mental health conditions [44,45], with no associated psychiatric adverse effects [46]. Most DAAs, including SOF/VEL, have shown a favorable interaction profile with Selective Serotonin Reuptake Inhibitors (SSRIs), antipsychotics, and mood stabilizers [47], although clinicians should still monitor for possible DDIs. Furthermore, the achievement of SVR with DAAs has been associated with improvements in mental well-being and stability of psychiatric symptoms during and after therapy [44], although neurocognitive deficits may persist despite viral recovery.

Currently, among DAAs, SOF/VEL represents a valuable option for complex psychiatric patients. Its advantages include pangenotypic coverage, short treatment duration, minimal psychiatric side effects, and a favorable drug interaction profile. Several studies have shown that SOF/VEL maintains high rates of SVR in populations with psychiatric comorbidities, with evidence from both registration trials and real-world data supporting its efficacy and safety [48,49]. Importantly, SOF/VEL demonstrated limited clinically significant interactions with most used psychotropic medications, reducing the risk of destabilizing psychiatric conditions or causing adverse effects [2,50].

Anyway, for HCV patients with neuropsychiatric disorders, establishing dedicated care pathways is crucial. Multidimensional clinical monitoring—by addiction, psychiatry, and hepatology specialists—may enable early detection of complications and support holistic care. Integrated approaches, including coordinated services and shared decision-making, can improve treatment alignment and satisfaction. In particular, the presence of psychiatric disorders represents a barrier to adherence to treatment. SOF/VEL offers the advantage of once-daily dosing, which helps to simplify therapy for such complex patients. On the other hand, treatment discontinuation is not uncommon in these patients. Retention in care should be enhanced, possibly through peer support, flexible scheduling, telemedicine, incentives, and stigma reduction. A summary of key findings and clinical implications is reported in Table 2.

### 4.3. Patients Who Use Substances or Are Being Treated with Opioid Agonists

The premise that global HCV elimination is fundamentally contingent on successfully treating PWUD is scientifically and epidemiologically sound. This population, particularly people who inject drugs (PWID), is consistently identified as the core reservoir of infection in Europe and worldwide, making them the primary group driving new transmissions [51]. Current guidelines (AASLD-IDSA) state that recent or active injection drug use is NOT a contraindication to HCV treatment [31]. Studies confirm that DAA therapies (which boast cure rates > 95%) in PWID, including those on opioid agonist therapy (OAT) or with active drug use, yield high SVR rates comparable to those in the general population [52]. The effectiveness of the treatments is well established. SVR rates achieved with DAAs in PWUD are comparable to those seen in other populations. DAAs continue to demonstrate high effectiveness in individuals receiving methadone or buprenorphine treatment [53,54,55]. The effectiveness of DAAs in PWUD is well known, provided that two key criteria are fulfilled: firstly, treatment involves the latest generation antiviral agents, and secondly, patient management takes place within dedicated addictive behavior services (SerDs). The same can be said about the safety of the treatment: DAAs have a favorable side effect profile (commonly mild headache/fatigue) and no consistent signal of increased adverse events emerged in PWUD or in persons on OAT [54,55]. Integrated care models are the preferred approach to treatment delivery. This means combining HCV treatment with other essential health services to which the patient may already have access. This integrated approach has demonstrated improved treatment persistence, leading to increased cure rates [56], as reported also by the ECDC (European Centre for Disease Prevention and Control) in its 2023 update on prevention and control of infectious diseases among PWID.

An Italian real-world analysis on the risk of DDIs in HCV patients treated with pangenotypic DAAs showed that PWUD, including those on OAT, can safely and effectively receive DAAs [57]. The SOF/VEL regimen was more commonly prescribed to older patients and those taking two or more additional medications with a potential for multiple DDIs, which supports its use in individuals with a greater burden of comorbidities and polypharmacy [57]. Clinical evidence and real-world experience indicate that PWUD, including those actively injecting or on opioid substitution therapy, can achieve high SVR with SOF/VEL when supported by adherence strategies and integrated care models [58]. Analyzing potential DDIs of DAAs with opioids (including fentanyl and oxycodone), no clinically significant interactions are expected. Prescribing information clarifies that SOF/VEL does not necessitate dose adjustments of these agents, and no clinically significant interactions are expected with antipsychotics (aripiprazole, quetiapine) either, which supports its coadministration in patients with comorbid psychiatric conditions. The lack of meaningful interaction of SOF/VEL with common antipsychotics is clinically helpful.

However, in case of polysubstance use, although direct pharmacokinetic interactions with many substances appear to be limited, counseling on dosing consistency, avoidance of missed doses, and recognition of adverse symptoms should be prioritized, especially when multiple medications are involved, and mental health should be monitored and continuity of care during treatment adequately supported [59].

A specific warning could be given related to oxycodone: the number of seizures in Italy has shown a marked overall increase over the last decade, rising from 355 cases in 2015 and peaking at 11,908 in 2023 despite notable fluctuations (e.g., 16,384 in 2020), a pattern that raises concern about potential clinical implications, including possible drug–drug interactions between oxycodone and direct acting antivirals (DAAs) [60].

## 5. Patients with Chronic HCV Infection and a Diagnosis of Cancer Requiring Therapy

The seroprevalence of HCV among cancer patients varies markedly according to the type of malignancy but generally exceeds that of the general population. In the United States as in the European Union, for patients with solid tumors other than hepatocellular carcinoma (HCC), the prevalence ranges from 1 to 5% up to 10.6%. However, in hematological cancers, such as lymphoma and leukemia, the prevalence can reach up to 30% [61]. CHC has been consistently associated with B-cell non-Hodgkin lymphoma. In countries with a high prevalence of the virus, it is estimated that CHC could account for up to 10% of B-cell non-Hodgkin lymphoma cases [62]. In Italy, it is estimated that 10% of lymphoma cases are attributable to HCV infection [63].

Recognizing and addressing HCV infection in cancer patients is crucial, as it may influence both cancer development and outcomes, and can affect decisions regarding cancer therapy and overall patient management. DAA therapy is highly effective, achieving an SVR in up to 92% of cases. Recent advancements have made DAAs more accessible and effective for patients with cancer, including those with hematological malignancies [62]. DAAs are considered the standard therapy for CHC-related cryoglobulinemia and for achieving remission in hematological malignancies, with low-grade B-cell non-Hodgkin lymphoma being a key example. For patients diagnosed with indolent lymphomas, especially marginal zone lymphoma, DAAs have demonstrated effectiveness in producing regression in roughly 50% of cases. As a result, DAAs are recommended as the first-line treatment for marginal zone lymphoma [62].

The approach to DAA therapy should be tailored depending on whether the treatment of the hematological malignancy is urgent or not. In the first case, the ECIL-9 recommends that DAAs should be given concomitantly with chemotherapy, whereas in the second case, HCV can be treated before chemotherapy [62]. However, unless the cancer is uncontrolled, antiviral therapy should generally be administered. Current evidence supports the benefits of DAAs in improving outcomes and survival in cancer patients. An accurate analysis of potential DDIs and a multidisciplinary approach involving oncologists and hepatologists is recommended to optimize timing and coordination of care for patients with both HCV and cancer (Table 3).

Therapy with DAAs in HCV patients with malignancies carries important benefits. A thorough meta-analysis has demonstrated that the incidence of HCC decreases over time after HCV eradication [64]. Further research highlights substantial advantages for patients with cirrhosis and Barcelona Clinic Liver Cancer (BCLC) stage 0/A HCC who have achieved a complete oncological response and subsequently attain SVR, including improved overall survival, lower rates of liver decompensation, and reduced HCC recurrence [65,66,67]. Moreover, a recent study found that achieving SVR with DAAs is linked to a decreased risk of liver-related death and enhanced liver function, even among those with HCC and advanced fibrosis or cirrhosis [68]. A very recent multicenter retrospective study demonstrated the safety and efficacy of DAAs in HCV patients with unresectable/advanced HCC treated with atezolizumab plus bevacizumab, showing that employing an integrated therapeutic strategy can maximize the effectiveness of systemic treatments, especially for those patients who may be suitable for conversion approaches [69].

When considering antiviral therapy in cancer patients with HCV infection, the potential for viral reactivation is a risk to consider. Reactivation rates can range between 5% and 10%, but such episodes are typically asymptomatic and may go unnoticed in clinical practice [70,71]. It is advisable to screen for chronic HBV and HCV infections and to implement regular monitoring of affected patients throughout the course of novel anticancer therapies.

There may be a risk of DDIs between antiviral agents and anticancer treatments, which necessitates careful management and may further complicate therapeutic decisions. In most cases, interactions are anticipated based on pharmacokinetic mechanisms, as surveillance data remain limited. Predictive DDI data is available for many antineoplastic drugs belonging to the class of mitotic spindle inhibitors, such as vincristine and vinblastine, or antimetabolites like 5FU, but also for molecularly targeted agents, including tyrosine kinase inhibitors, Janus kinase inhibitors, BCL2 inhibitors, and PARP protein blockers, drugs from the hormone class, such as aromatase inhibitors or androgen inhibitors, and antitumor antibiotics like doxorubicin. The drugs listed above are responsible for DDIs, with the most frequent interactions involving agents metabolized by CYP3A4 (see tyrosine kinase inhibitors) (Liverpool HEP interactions database https://www.hep-druginteractions.org, accessed on 3 August 2026). Most hematological drugs and biologics share a very low potential for DDIs and can usually be used concurrently with anti-HCV drugs [61]. Currently, several cancers are treated with immune checkpoint inhibitors (ICPIs). These types of cancer do not seem to be associated with inferior efficacy and have shown a low reactivation rate. No significant side effects have been reported in real-world data, either with SOF/VEL or glecaprevir/pibrentasvir, in patients with HCV-related HCC treated with AtezoBev [69], as well as with sofosbuvir/ledipasvir in HCV patients with large B-cell non-Hodgkin’s lymphoma [72].

Regarding the choice of the pangenotypic regime to be used, it can be stated that SOF/VEL is used in significantly higher proportions in patients starting therapy [73]. In a prospective observational study in 159 HCV patients with different solid and hematological malignancies treated with sofosbuvir-based regimens, SVR12 was achieved in 91% of patients. Overall, these regimens were effective and well-tolerated (with only mild adverse events); they may permit access to investigational cancer therapy, thus expanding treatment options, and may induce remission of non-Hodgkin lymphoma [74].

**Table 3:** Summary of recommendations for managing cancer patients with HCV and clinically relevant DDIs.

Type of Cancer	Expert Opinion	DDI Key Findings	Clinical Implication
Patients with solid cancer	Careful selection of pangenotypic regimen is crucial in patients with solid tumor receiving cancer treatment.	CYP3A4 is a key liver enzyme for taxmen, TKIs, and vinca alkaloids. Protease inhibitors interacting with CYP3A4 increase toxicity of these antineoplastic drugs (Liverpool HEP interactions)	A multidisciplinary approach involving liver disease specialists in the management of oncologic patients with HCV infection may help in avoiding treatments, including drugs at risk of interaction with CYP3A4
Patients with B-cell lymphoma	DAAs can be considered the standard therapy for CHC-related cryoglobulinemia and for low-grade B-cell non-Hodgkin lymphoma. Treatment of HCV infection in these patients improves outcomes and survival.	The existing literature supports concomitant administration of DAAs and chemotherapy when needed [72]	A multidisciplinary approach to hematologic cancer helps in selecting pangenotypic regimens not containing PIs and reduces the risk of DDIs
Patients with HCC	Patients with HCC and active HCV infection require treatment. Achieving HCV clearance improves outcomes and survival.	Several cancers including HCC are currently treated with ICI. Studies on the combination of atezolizumab/bevacizumab and pangenotypic regimens are available and suggest high efficacy. No safety issues were reported [69]	In patients with HCC, pangenotypic regimens have similar efficacy to the general population. No DDIs are expected with regimens not containing PIs

TKIs, tyrosine kinase inhibitors; HCV, hepatitis C virus; CHC, chronic hepatitis C; DAAs, direct-acting antivirals; PIs, protease inhibitors; HCC, hepatocellular carcinoma; ICI, immune checkpoint inhibitor; DDIs, drug–drug interactions.

## 6. Transplant Patients

The management of HCV infection in transplantation has undergone a profound transformation with the advent of DAAs. Historically, organs from HCV-infected donors were frequently declined because of transmission risk and inferior post-transplant outcomes. However, successful solid organ transplants from HCV-infected deceased donors have now been widely reported [75], reflecting a paradigm shift enabled by highly effective antiviral therapies.

### 6.1. Prevalence of HCV in Donors and Recipients

In the United States, the prevalence of HCV among solid organ donors has increased in recent years, largely due to the opioid epidemic and rising injection drug use. Data from the Organ Procurement and Transplantation Network (OPTN) and the Scientific Registry of Transplant Recipients (SRTR) indicate that the proportion of deceased donors with any HCV marker increased from 6.2% in early 2015 to 7.4% in late 2017, with a concomitant rise in HCV-positive donors [76,77]. In liver transplantation, HCV-positive donor prevalence rose from 3% in 1995 to nearly 10% in 2013 and has further expanded in the DAA era [77]. Globally, current prevalence among US donors is estimated at 6–10%, with lower but variable rates in other countries depending on local epidemiology.

Among solid organ transplant recipients, HCV seropositivity remains clinically significant. A recent SRTR analysis (2006–2021) reported HCV seropositivity in 5.0% of kidney, 2.2% of heart, and 2.2% of lung transplant recipients in the United States [78].

In hematopoietic stem cell transplantation (HSCT), HCV prevalence is lower, approximately 1–4% in Europe and the United States [62]. Although untreated HCV may lead to long-term hepatic complications, short-term HSCT outcomes are not significantly worsened when infection is appropriately managed.

### 6.2. Transplantation from HCV-Positive Donors to HCV-Negative Recipients

In Italy, the management of solid organ transplantation from HCV-infected donors—including which donors may be considered acceptable and how recipients are managed—is guided by evolving national policy from the National Transplant Centre (CNT/AIFA) together with technical recommendations that reflect the availability of highly effective DAAs. However, there is no CNT circular dedicated exclusively to HSCT, like the recent one for solid organs. Italian centers align with EBMT (European Society for Blood and Marrow Transplantation) and WBMT (Worldwide Network for Blood and Marrow Transplantation) recommendations.

The introduction of DAAs has enabled the safe transplantation of organs from HCV-infected donors into HCV-negative recipients. This strategy reduces waiting lists and mortality, and utilizes organs previously discarded, often from younger donors [79]. AASLD/IDSA guidelines recommend DAA treatment for all HCV-infected solid organ transplant recipients, including those receiving viremic grafts [31].

Clinical trials and real-world studies demonstrate SVR rates approaching 100%, with graft and patient survival comparable to recipients of HCV-negative organs [55,79,80]. Early or pre-emptive therapy is preferred to minimize complications such as hepatitis or glomerulonephritis. In non-liver recipients, short DAA courses started in the peri-transplant period may be sufficient, whereas liver transplant recipients generally require 12-week regimens [31,55].

### 6.3. Treatment of HCV Infection in Transplant Recipients

Preferred pangenotypic regimens include sofosbuvir/velpatasvir (SOF/VEL) and glecaprevir/pibrentasvir, with SVR12 rates of 96–98% and excellent tolerability [31,81,82]. In a retrospective cohort study in HCV-negative recipients receiving kidney transplantation from HCV-positive donors, SOF/VEL pre- and post-transplantation treatment has proven safe and effective [83]. Although SOF/VEL may rarely cause bradycardia when coadministered with amiodarone, clinical experience in heart and lung transplant recipients suggests manageable and transient events [84].

In HSCT, EBMT and ECIL-9 guidelines recommend pangenotypic DAAs either before or after transplantation, depending on urgency and DDI profiles [61,85,86]. Studies confirm SVR rates exceed 90%, without increased graft-versus-host disease or transplant-related toxicity [87,88]. Long-term liver surveillance remains necessary in patients with advanced fibrosis. It can be concluded that pangenotypic DAA therapy is the standard of care for HCV infection in HSCT recipients, with regimen selection and timing individualized according to clinical context and DDI profile.

### 6.4. Drug–Drug Interactions

Management of DDIs is crucial in transplant recipients. NS3/4A protease inhibitors (e.g., glecaprevir, grazoprevir) interact significantly with calcineurin inhibitors (tacrolimus, cyclosporine) and mTOR inhibitors (sirolimus, everolimus), necessitating close therapeutic drug monitoring [31,62,89]. Certain combinations are contraindicated (e.g., elbasvir/grazoprevir with cyclosporine; high-dose cyclosporine with glecaprevir/pibrentasvir). NS5A/NS5B inhibitors generally have fewer interactions but still require monitoring, particularly as hepatic function improves after SVR [55,90]. Table 4 summarizes the main findings in the context of transplantation for patients with HCV and their clinical implications.

## 7. Hormones and DAAs

### 7.1. Women of Childbearing Potential

It has been estimated that 21% of global HCV cases occur in women of childbearing age [91]. For women of reproductive age, special attention to viral eradications is warranted due to the potential risk of transmitting HCV from mother to child. Among pregnant women with HCV, about 3–6% of their newborns will contract the virus [92]. Roughly one in five children who acquire HCV at birth will spontaneously eliminate the infection, whereas around 30% develop chronic liver disease [93]. DAAs offer women with hepatitis C a treatment that is safe, effective, well-tolerated, and capable of curing the infection. Evidence indicates that DAA therapy achieves even higher rates of effectiveness in women compared to men, consistently across all ages until the onset of menopause, when treatment efficacy is at its lowest among women [94]. Because of their favorable efficacy and safety profile, DAAs are seen as promising candidates for reducing perinatal transmission of HCV, although they are not currently recommended during pregnancy, primarily due to limited data on their pharmacokinetics and safety in this population. However, early research indicates DAAs may be highly effective and pose minimal risk for adverse events in pregnant women. Research on the pharmacokinetics, safety, and effectiveness of SOF/VEL in pregnant women with hepatitis C found that drug levels during pregnancy were comparable to those in non-pregnant populations, indicating that further investigation into the use of SOF/VEL during pregnancy is warranted [95,96]. As additional pharmacokinetic and epidemiological evidence emerges, DAAs may become the preferred therapy for managing HCV in pregnancy and preventing mother-to-child transmission of hepatitis C.

### 7.2. Women Taking Contraceptives

A study evaluated the potential for a DDI between HCV direct-acting antivirals sofosbuvir or ledipasvir and oral hormonal contraceptives. Pharmacokinetic parameters were similar to historical data. Pharmacodynamic markers, luteinizing hormone, follicle-stimulating hormone, and progesterone values were generally comparable in all cycles. Therefore, using sofosbuvir-based treatments alongside oral contraceptives containing norgestimate and ethinyl estradiol is not expected to reduce contraceptive effectiveness [97]. SOF/VEL, according to the Interaction Checker of the Liverpool University (HEP Drug Interactions), has no significant interaction expected with desogestrel, dienogest, drospirenone, estradiol, levonorgestrel, levonorgestrel/ethinylestradiol, desogestrel/ethinylestradiol, drospirenone/estradiol, drospirenone/ethinylestradiol, gestodene/ethinylestradiol, and norgestrel/ethinylestradiol.

### 7.3. Transgender Individuals

The terms transgender and gender-diverse refer to people whose gender identity does not match the sex they were assigned at birth. Transitioning is the process of modifying one’s social, physical, or legal characteristics to better align with their gender identity, which can include changes in name, pronouns, and appearance, or medical interventions. A significant number of transgender individuals pursue hormone therapy as an integral part of their transition. Transgender individuals often encounter a range of health, social, and medical challenges. These challenges can increase their vulnerability to several infections, particularly sexually transmitted and bloodborne diseases (STBBIs) such as hepatitis C, hepatitis B, and HIV/AIDS [98]. The overall prevalence of HCV among all transgender populations globally has been estimated at 9%, while the corresponding prevalence among male-to-female transgender people has been estimated at 5%. Among American transgender people, these estimates are around 10%. All these prevalence data are higher than those in the general population [99]. In Italy, in a consecutive series of 700 transgender individuals, the serological prevalence of HCV was found to be 5.6%, again higher than that reported in the general population [100].

In treating transgender people with HCV infection, special attention should be paid to possible interactions between antiviral treatment and hormone therapy, especially in transwomen [101]. In order to deliver safe and supportive medical treatment, clinicians must recognize and appropriately manage any potential DDIs between gender-affirming hormones and other prescribed therapies. Gender-affirming hormonal therapy may be involved in pharmacokinetic and/or pharmacodynamic DDIs with DAAs, among other antiviral medications. Glecaprevir/pibrentasvir, SOF/VEL, and SOF/VEL/voxilaprevir have the potential to raise estradiol levels by inhibiting organic anion transport protein 1B1. However, it remains uncertain whether this effect is significant enough to be of clinical importance [101]. Velpatasvir, ledipasvir, elbasvir, and sofosbuvir do not induce or inhibit CYP enzymes. On the other hand, all DAAs have potential pharmacokinetic interactions with androgen blockers and adjunctive therapies, and testosterone [102].

## 8. Conclusions

Managing multiple medications in HCV patients with comorbidities poses a significant challenge, as the potential for DDIs can undermine both the effectiveness and safety of antiviral treatments. DAAs have dramatically improved the management of HCV patients; however, they have different DDI profiles. SOF/VEL has been shown to be associated with minimal clinically relevant interactions and offers a simple dosing regimen. This offers practical advantages within integrated care settings, where multidisciplinary teams are tasked with overseeing HCV management alongside psychiatric, metabolic, and renal disorders.

In patients with cardiometabolic disease, DAAs offer the potential for cure in HCV patients, but their safe use depends on thorough medication review, proactive monitoring, and coordinated multidisciplinary care. Treatment with SOF/VEL showed to have a favorable impact on glycemic control and was generally well-tolerated. Anticipating metabolic changes after eradication, treating DDIs as a main clinical endpoint, and embedding antiviral therapy within cardiology and diabetology pathways transform HCV treatment into a platform for simplifying care, improving adherence, and reducing preventable complications in this vulnerable population.

In patients with HCV, there is a high prevalence of CKD due to advanced age and polypharmacy for multiple coexisting health conditions. Initially sofosbuvir-based antiviral regimens were used with caution in patients with eGFR < 30 mL/min, due to fear of accumulation of SOF metabolites. However, subsequent clinical studies have provided strong evidence on the efficacy and safety of sofosbuvir-containing regimens in patients with advanced CKD, including those on dialysis.

Chronic HCV infection is associated with a substantial and multifactorial neuropsychiatric burden, in which biological, psychological, and social determinants interact to negatively influence both mental health and treatment outcomes. The introduction of DAAs—particularly SOF/VEL—has transformed the therapeutic landscape, offering a highly effective, well-tolerated, and accessible option even for patients with complex psychiatric comorbidities. Importantly, viral eradication with DAAs is increasingly linked to improvements in psychological well-being, although residual cognitive and psychiatric challenges may persist, underscoring the need for ongoing multidisciplinary care.

Therapy with DAAs in HCV patients with malignancies carries important benefits. Several anticancer drugs are responsible for DDIs. Therefore, DDIs between DAAs and cancer therapies are perceived as a barrier to HCV treatment in persons with cancers. However, DDIs with DAAs are mostly related to agents metabolized by CYP3A4. No significant side effects have been reported in the real world, either with SOF/VEL or glecaprevir/pibrentasvir, in patients with HCV-related HCC treated with AtezoBev.

The advent of DAAs has also revolutionized the management of HCV in transplant settings, enabling the safe use of organs from HCV-infected donors with outcomes comparable to HCV-negative grafts. Early or pre-emptive antiviral therapy, together with careful management of DDIs, represents the cornerstone of optimizing both graft and patient survival.

Treatment with SOF/VEL is well-tolerated even in PWUD and PWID and has similar efficacy to the general population. The drug is particularly suitable with opioid agonist therapies. One of the essential elements for achieving this goal remains the possibility of integrated treatment within addiction services.

For women using contraceptives, SOF/VEL is not expected to have any significant interactions with the most commonly used contraceptive agents, according to the HEP Drug Interactions. Therefore, contraceptive effectiveness should not be compromised. This is particularly relevant when managing HCV infection in the transgender population, who are at increased risk for parenteral infections. In transwomen especially, the absence of interactions with sex hormones means that anti-HCV treatment can proceed safely. However, there is limited data regarding DDIs between SOF/VEL and androgen blockers, adjunctive therapies, or testosterone.

DAAs offer the opportunity not only to cure HCV but also to improve the course of comorbidities, provided that DDIs are no longer considered mere minor details. In these comorbid populations, safety comes from planning, not hope.

## 9. Expert Opinion

- In patients with diabetes and cardiovascular disease, DAAs should be prescribed using a standardized cardiometabolic safety workflow, not as a purely hepatology-driven prescription, based on three pillars:
  (i) Anticipate pharmacodynamic shifts in glycemia, intensifying self-monitoring for insulin-treated patients (mandatory) for those on sulfonylureas or complex regimens (strongly advisable); dose down-titration should be proactive rather than reactive; for patients already at risk (older age, advanced liver disease, recurrent hypoglycemia, low reserve), antidiabetic therapy should be simplified toward low-hypoglycemia-risk options.
  (ii) Treat cardiovascular DDIs as a primary endpoint; statins, antiarrhythmics, antiplatelets, and anticoagulants are common in this population, and minor DDIs become major when layered onto renal dysfunction, cirrhosis, and variable adherence: a structured medication reconciliation at baseline should include explicit documentation of the DDI assessment and a monitoring plan. After SVR, lipid changes are expected and should not be ignored, so a post-treatment lipid panel should be considered part of standard cardiovascular stewardship, with statin optimization based on overall risk and tolerance
  (iii) Identify and manage high-severity, low-frequency interactions. The sofosbuvir–amiodarone bradyarrhythmia risk, although uncommon, has too serious consequences for casual management. If amiodarone is present, the plan should be shared with cardiology and substitution considered whenever feasible. If not feasible, monitoring must be arranged deliberately, not improvised.
- For atrial fibrillation patients on DOACs, continuing anticoagulation during DAA therapy is often reasonable, but only within a disciplined framework: verify regimen compatibility, reassess renal and hepatic function, minimize concurrent bleeding enhancers (NSAIDs, unnecessary antiplatelets), and intensify monitoring for bleeding during therapy.
- In HCV patients with CKD, since achieving a SVR has been shown to reduce the risk of ESRD-related complications, improve extra-hepatic symptoms, and significantly improve survival outcomes, sofosbuvir-based regimens should be considered as a first-line treatment option.
- To effectively manage HCV patients with psychiatric comorbidities, integrated, multidisciplinary care models are essential to ensure both virological success and mental health support. SOF/VEL represents a particularly suitable therapeutic option in this population due to its favorable safety profile, limited drug–drug interactions, and simplified dosing regimen. Future strategies should prioritize retention in care and adherence through patient-centered interventions, including telemedicine, peer support, and stigma reduction initiatives.
- Current evidence supports the benefits of DAAs in improving outcomes and survival in patients with either HCV-associated or non-HCV-associated cancer and active HCV infection. Therefore, antiviral treatment is recommended for all, except those with a short life expectancy that cannot be remediated by HCV therapy. DAA regimens are effective and well-tolerated in people with cancer. Despite limited published data, avoiding drugs metabolized by CYP3A4 reduces the risk of potential adverse events related to the concomitant antiviral regimen.
- The expanding use of HCV-viremic donors should be considered a strategic opportunity to reduce organ shortage, particularly when supported by timely initiation of pangenotypic DAA therapy. Future efforts should focus on refining individualized treatment strategies based on transplant type, timing, and DDI profiles to further improve outcomes. Early or pre-emptive antiviral therapy is crucial to optimize both graft and patient survival. Long-term follow-up, especially in patients with advanced liver disease, remains essential to fully assess the impact of this evolving paradigm on post-transplant morbidity and survival.
- For treating PWUD and PWID with HCV, DAAs are effective and well-tolerated. An integrated treatment within addiction services is strongly recommended to optimize outcomes.
- SOF/VEL has shown to be safe in women taking contraceptives and in transwomen. Caution should be exercised in transmen taking androgen blockers and adjuvant therapies, and testosterone, due to potential DDIs with DAAs.

## Figures and Tables

**Table 1:** Summary of clinically relevant DAA interactions with antidiabetic and cardiovascular drugs.

Drug Class	Key Findings	Clinical Implications
Antidiabetic drugs (insulin, sulfonylureas, metformin, DPP-4 inhibitors, GLP-1 receptor agonists, SGLT2 inhibitors)	After DAA-induced SVR, glycemic control may improve (HbA1c/fasting glucose reduction), but the magnitude and durability are variable. The main practical issue is pharmacodynamic: improved hepatic function and reduced inflammation-driven insulin resistance may increase hypoglycemia risk, especially with insulin and sulfonylureas [19,20,21,22,23,24].	Intensify glucose self-monitoring during the first weeks of DAA therapy and early post-treatment. Promptly reassess and down-titrate insulin/sulfonylureas when appropriate. Avoid the false assumption of “resolved diabetes”; ensure structured long-term metabolic follow-up.
Lipid-lowering drugs (statins, ezetimibe, others)	Post-SVR, total and LDL cholesterol often increase, likely reflecting reversal of virus-associated hypolipidemia rather than a direct harmful drug effect [34,35]. Potential DDIs may require temporary statin dose adjustment or discontinuation depending on the DAA regimen and the specific statin [37].	Obtain a post-treatment lipid panel (commonly around SVR12 in routine practice). Optimize lipid-lowering therapy according to global cardiovascular risk. During DAA therapy, review the DDI profile and adjust statin choice/dose when needed.
Anticoagulants/antithrombotics (DOACs, VKAs; antiplatelets as co-therapy)	DDI risk is mechanistically plausible via transporters/enzymes affecting DOAC exposure [38,39]; real-world data suggest coadministration is often feasible with appropriate selection and monitoring [40]. Bleeding risk is amplified by cirrhosis-related factors (thrombocytopenia, varices) and comedications (antiplatelets, NSAIDs).	Before DAAs: formal medication reconciliation and DDI check. During therapy: enhanced bleeding surveillance and periodic labs as clinically indicated. If on warfarin, plan more frequent INR monitoring and dose adjustment as needed. Minimize avoidable bleeding enhancers (NSAIDs, unnecessary antiplatelets).
Antiarrhythmic drugs (focus on amiodarone)	Rare but potentially severe bradyarrhythmia has been reported with sofosbuvir-containing regimens when coadministered with amiodarone [41]. This represents high-severity, low-frequency interaction.	If amiodarone is present: cardiology co-management is strongly advised. Consider substitution when feasible; if not feasible, implement planned ECG/heart-rate monitoring. Educate the patient on bradycardia symptoms and ensure rapid access to evaluation.

Abbreviations: DAA, direct-acting antiviral; SVR, sustained virologic response; DOAC, direct oral anticoagulant; INR, international normalized ratio; NSAIDs, non-steroidal anti-inflammatory drugs; VKA, vitamin K antagonist.

**Table 2:** Patients with HCV and neuropsychiatric disorders: summary of key findings and clinical implications.

Domain	Key Findings	Clinical Implications
HCV prevalence	High rates of depression (30–50%), anxiety, cognitive impairment, substance use [42].	Screen routinely; address psychosocial factors
DAA therapy	High efficacy, safe, with no significant psychiatric adverse effects [44,45,46]; SOF/VEL well-tolerated [47,48,49].	First-line use; suitable for psychiatric patients; SOF/VEL is a preferred option due to simplicity and safety
Treatment timing	Early treatment feasible and beneficial regardless of psychiatric status; SVR improves mental well-being [44].	Do not delay treatment; timely initiation can improve both hepatic and psychological outcomes
Drug–drug interactions	DAAs generally have a favorable interaction profile with psychotropics [47].	Monitor DDIs; treatment is generally manageable without major adjustments
Follow-up	Risk of poor adherence, discontinuation; multidisciplinary care improves retention.	Multidisciplinary care; supportive strategies (telemedicine, peer support) should be implemented

DAA, direct-acting antiviral; SOF/VEL, sofosbuvir/velpatasvir; DDIs, drug–drug interactions.

**Table 4:** Main findings in the context of transplantation for patients with HCV and their clinical implications.

Domain	Key Findings	Clinical Implications
HCV prevalence in donors	6–10% in the United States, increasing due to injection drug use [76,77].	Need of protocols for management of HCV infection in uninfected donors from HCV RNA-positive recipients
HCV prevalence in recipients	Kidney 5%, heart/lung 2.2% [78].	HCV remains clinically relevant in transplant populations
HSCT setting	Prevalence 1–4% [62].	No significant impact on short-term outcomes when appropriately managed
HCV-positive donors to HCV-negative recipients	With post-transplant HCV treatment SVR rates ~100%, comparable survival outcomes [55,79,80].	Expanded availability of transplantable organs; reduced waiting list times and mortality
DAA therapy	SOF/VEL, GLE/PIB (SVR 96–98%) [31,81,82].	Established standard of care with excellent tolerability
Treatment timing	Early or pre-emptive therapy preferred [31].	Minimizes post-transplant complications
DDIs	Significant interactions with immunosuppressants [31,62,89].	Requires careful monitoring and dose adjustments
Follow-up	Essential in patients with advanced fibrosis [86].	Prevention and early detection of long-term complications

HCV, hepatitis C virus; HSCT, hematopoietic stem cell transplantation; SVR, sustained viral response; DAA, direct-acting antiviral; SOF/VEL, sofosbuvir/velpatasvir; GLE/PIB, glecaprevir/pibrentasvir; DDIs, drug–drug interactions.

## Data Availability

No new data were created or analyzed in this study. Data sharing is not applicable to this article.

## References

[1] (2020). European Association for the Study of the Liver; Clinical Practice Guidelines Panel: Chair; EASL Governing Board representative; Panel members. EASL recommendations on treatment of hepatitis C: Final update of the series. J. Hepatol..

[2] Pugliese N., Conti F., Rosato V., Gallo P., Gitto S., Riglietta M., Frigerio F., Perrone V., Veronesi C., Cappuccilli M. (2025). Epidemiologic Characteristics Determining the Choice of Direct-Acting Antiviral Therapy in HCV Patients: An Italian Real-World Evidence Study. Pathogens.

[3] Behera M.K., Majji P., Behera S., Pani M., Mohapatro A., Patra U.C., Jena S.K. (2024). The Fixed Dose Combination of Sofosbuvir and Velpatasvir is Safe and Effective in Patients of Chronic Hepatitis C with End-stage Renal Disease. J. Clin. Exp. Hepatol..

[4] Xiao J., Zhang X., Mao X., Lai S., Li S., Sheng Y. (2025). Efficacy and Safety of Velpatasvir Plus Sofosbuvir with or Without Ribavirin in Hepatitis C Patients with Decompensated Cirrhosis: A Systematic Review and Meta-Analysis. J. Viral Hepat..

[5] Nawaz R., Ahmad M., Raza M.S., Rashad M., Nawaz A., Tabassum K., Hassan J.U., Ahad A., Idrees M. (2024). Coincidence of HCV and chronic kidney disease-a systematic review and meta-analysis. BMC Public Health.

[6] Khan M.U., Mahmoud M.I., Butt A.A. (2020). Hepatitis c virus and chronic kidney disease. Expert Rev. Gastroenterol. Hepatol..

[7] Awan A.A.Y., Berenguer M.C., Bruchfeld A., Fabrizi F., Goldberg D.S., Jia J., Kamar N., Mohamed R., Pessôa M.G., Pol S. (2023). Prevention, Diagnosis, Evaluation, and Treatment of Hepatitis C in Chronic Kidney Disease: Synopsis of the Kidney Disease: Improving Global Outcomes 2022 Clinical Practice Guideline. Ann. Intern. Med..

[8] De Nicola S., Aghemo A. (2016). The quest for safe and effective treatments of chronic hepatitis C in patients with kidney impairment. Liver Int..

[9] Li M., Chen J., Fang Z., Li Y., Lin Q. (2019). Sofosbuvir-based regimen is safe and effective for hepatitis C infected patients with stage 4-5 chronic kidney disease: A systematic review and meta-analysis. Virol. J..

[10] Fabrizi F., Cerutti R., Dixit V., Ridruejo E. (2021). Sofosbuvir-based regimens for HCV in stage 4-stage 5 chronic kidney disease. A systematic review with meta-analysis. Nefrol. (Engl. Ed.).

[11] Solitano V., Aghemo A. (2021). Sofosbuvir in HCV patients with chronic kidney disease: No time for caution. Liver Int..

[12] Huang C.F., Tseng K.C., Cheng P.N., Hung C.H., Lo C.C., Peng C.Y., Bair M.J., Yeh M.L., Chen C.H., Lee P.L. (2022). Impact of Sofosbuvir-Based Direct-Acting Antivirals on Renal Function in Chronic Hepatitis C Patients with Impaired Renal Function: A Large Cohort Study From the Nationwide HCV Registry Program (TACR). Clin. Gastroenterol. Hepatol..

[13] De A., Roy A., Verma N., Mishra S., Premkumar M., Taneja S., Singh V., Duseja A. (2022). Sofosbuvir plus velpatasvir combination for the treatment of chronic hepatitis C in patients with end stage renal disease on renal replacement therapy: A systematic review and meta-analysis. Nephrology.

[14] Taneja S., Duseja A., Mehta M., De A., Verma N., Premkumar M., Dhiman R.K., Singh V., Singh M.P., Ratho R.K. (2021). Sofosbuvir and Velpatasvir combination is safe and effective in treating chronic hepatitis C in end-stage renal disease on maintenance haemodialysis. Liver Int..

[15] Liu C.H., Chen C.Y., Su W.W., Tseng K.C., Lo C.C., Liu C.J., Chen J.J., Peng C.Y., Shih Y.L., Yang S.S. (2022). Sofosbuvir/velpatasvir with or without low-dose ribavirin for patients with chronic hepatitis C virus infection and severe renal impairment. Gut.

[16] Shahid S., Asghar S., Mahmood T., Fatima M., Rasheed A., Asghar S. (2023). Sofosbuvir and Velpatasvir Regimen Outcome for Chronic Hepatitis C Patients with End-Stage Renal Disease Undergoing Hemodialysis. Cureus.

[17] Mohammad N., Khan D. (2024). Efficacy of Sofosbuvir and Velpatasvir Combination in the Treatment of Hepatitis C Virus (HCV) in Chronic Kidney Disease (CKD) Patients. Cureus.

[18] Giannini E.G., Mangia A., Morisco F., Toniutto P., Avogaro A., Fagiuoli S., Borghi C., Frigerio F., Nugnes M., Veronesi C. (2025). A Real-World Analysis of the Population with Hepatitis C Virus Infection Affected by Type 2 Diabetes in Italy: Patients’ Characteristics, Comorbidity Profiles and Treatment Patterns. Medicina.

[19] Ciancio A., Bosio R., Bo S., Pellegrini M., Sacco M., Vogliotti E., Fassio G., Bianco Mauthe Degerfeld A.G.F., Gallo M., Giordanino C. (2018). Significant improvement of glycemic control in diabetic patients with HCV infection responding to direct-acting antiviral agents. J. Med. Virol..

[20] Gilad A., Fricker Z.P., Hsieh A., Thomas D.D., Zahorian T., Nunes D.P. (2019). Sustained Improvement in Type 2 Diabetes Mellitus is Common After Treatment of Hepatitis C Virus with Direct-acting Antiviral Therapy. J. Clin. Gastroenterol..

[21] Chen B., Iqbal U., Desai S.K., Gries J., Verheyen E., Xie M., El Halabi M., Gaines S., Weisberg I. (2024). The role of treatment of hepatitis C with direct-acting antiviral agents on glycaemic control in diabetic patients: An updated systematic review and meta-analysis. J. Viral Hepat..

[22] Mada P.K., Malus M.E., Parvathaneni A., Chen B., Castano G., Adley S., Moore M., Hieda M., Alam M.J., Feldman M. (2020). Impact of Treatment with Direct Acting Antiviral Drugs on Glycemic Control in Patients with Hepatitis C and Diabetes Mellitus. Int. J. Hepatol..

[23] Cacciola I., Russo G., Filomia R., Pitrone C., Caccamo G., Giandalia A., Alibrandi A., Stella Franzè M., Porcari S., Maimone S. (2021). Over time evaluation of glycaemic control in direct-acting antiviral-treated hepatitis C virus/diabetic individuals with chronic hepatitis or with cirrhosis. Liver Int..

[24] Tang J., Zhou J., Zhu L., Zhao Y., Yin G., Zhang R. (2025). Direct-acting antiviral treatment impact on glycemic control in HCV-infected T2DM patients: An observational study with Mendelian randomization analysis. Medicine.

[25] Li J., Gordon S.C., Rupp L.B., Zhang T., Trudeau S., Holmberg S.D., Moorman A.C., Spradling P.R., Teshale E.H., Boscarino J.A. (2019). Sustained virological response does not improve long-term glycaemic control in patients with type 2 diabetes and chronic hepatitis C. Liver Int..

[26] Russo F.P., Zanetto A., Gambato M., Bortoluzzi I., Al Zoairy R., Franceschet E., De Marchi F., Marzi L., Lynch E.N., Floreani A. (2020). Hepatitis C virus eradication with direct-acting antiviral improves insulin resistance. J. Viral Hepat..

[27] Zhou Y., Xie W., Zheng C., Liu L., Chen Z., Wang X. (2022). Hypoglycemia associated with direct-acting anti-hepatitis C virus drugs: An epidemiologic surveillance study of the FDA adverse event reporting system (FAERS). Clin. Endocrinol..

[28] Shimono Y., Enomoto H., Aizawa N., Takashima T., Ikeda N., Yuri Y., Fujiwara A., Yoshihara K., Yoshioka R., Kawata S. (2022). Possible Alterations in Appetite-related Molecules After the Elimination of Hepatitis C Virus. In Vivo.

[29] Wang S.J., Chang T.S., Lo C.C., Hung C.H., Huang C.W., Chong L.W., Cheng P.N., Bair M.J., Yeh M.L., Peng C.Y. (2025). Effects of Sofosbuvir/Velpatasvir Therapy on Extrahepatic Manifestations in Patients with Type 2 Diabetes and Chronic Hepatitis C: Insights from a Nationwide Hepatitis C Virus Registry in Taiwan. Kaohsiung J. Med. Sci..

[30] Lee K.K., Stelzle D., Bing R., Anwar M., Strachan F., Bashir S., Newby D.E., Shah J.S., Chung M.H., Bloomfield G.S. (2019). Global burden of atherosclerotic cardiovascular disease in people with hepatitis C virus infection: A systematic review, meta-analysis, and modelling study. Lancet Gastroenterol. Hepatol..

[31] Bhattacharya D., Aronsohn A., Price J., Lo Re V., AASLD-IDSA HCV Guidance Panel (2023). Hepatitis C Guidance 2023 Update: AASLD-IDSA Recommendations for Testing, Managing, and Treating Hepatitis C Virus Infection. Clin. Infect. Dis..

[32] Sasso F.C., Pafundi P.C., Caturano A., Galiero R., Vetrano E., Nevola R., Petta S., Fracanzani A.L., Coppola C., Di Marco V. (2021). Impact of direct acting antivirals (DAAs) on cardiovascular events in HCV cohort with pre-diabetes. Nutr. Metab. Cardiovasc. Dis..

[33] Calvaruso V., Petta S., Cacciola I., Cabibbo G., Cartabellotta F., Distefano M., Scifo G., Di Rosolini M.A., Russello M., Prestileo T. (2021). Rete Sicilia Selezione Terapia—HCV (RESIST-HCV). Liver and cardiovascular mortality after hepatitis C virus eradication by DAA: Data from RESIST-HCV cohort. J. Viral Hepat..

[34] Villani R., Di Cosimo F., Romano A.D., Sangineto M., Serviddio G. (2021). Serum lipid profile in HCV patients treated with direct-acting antivirals: A systematic review and meta-analysis. Sci. Rep..

[35] Gitto S., Cicero A.F.G., Loggi E., Giovannini M., Conti F., Grandini E., Guarneri V., Scuteri A., Vitale G., Cursaro C. (2018). Worsening of Serum Lipid Profile after Direct Acting Antiviral Treatment. Ann. Hepatol..

[36] Özdoğan O., Yaraş S., Ateş F., Üçbilek E., Sezgin O., Altıntaş E. (2020). The impact of direct-acting antiviral treatment on lipid metabolism and insulin resistance in chronic hepatitis C patients: Temporary? Permanent?. Turk. J. Gastroenterol..

[37] Smolders E.J., Ter Horst P.J.G., Wolters S., Burger D.M. (2019). Cardiovascular Risk Management and Hepatitis C: Combining Drugs. Clin. Pharmacokinet..

[38] Rosato V., Nevola R., Dallio M., Di Micco P., Spinetti A., Zeneli L., Ciancio A., Milella M., Colombatto P., D’Adamo G. (2024). Safety of Sofosbuvir-Based Direct-Acting Antivirals for Hepatitis C Virus Infection and Direct Oral Anticoagulant Co-Administration. J. Clin. Med..

[39] Canonico M.E., Sanna G.D., Siciliano R., Scudiero F., Esposito G., Parodi G. (2022). Drug-drug interactions between antithrombotics and direct-acting antivirals in hepatitis C virus (HCV) patients: A brief, updated report. Front. Pharmacol..

[40] McDaniel K., Utz A.E., Akbashev M., Fuller K.G., Boyle A., Davidson K., Marra F., Shah S., Cartwright E.J., Arora A.A. (2022). Safe co-administration of direct-acting antivirals and direct oral anticoagulants among patients with hepatitis C virus infection: An international multicenter retrospective cohort study. J. Viral Hepat..

[41] Caldeira D., Rodrigues F.B., Duarte M.M., Sterrantino C., Barra M., Gonçalves N., Pinto F.J., Ferreira J.J., Costa J. (2018). Cardiac Harms of Sofosbuvir: Systematic Review and Meta-Analysis. Drug Saf..

[42] Yarlott L., Heald E., Forton D. (2017). Hepatitis C virus infection, and neurological and psychiatric disorders—A review. J. Adv. Res..

[43] Yeoh S.W., Holmes A.C.N., Saling M.M., Everall I.P., Nicoll A.J. (2018). Depression, fatigue and neurocognitive deficits in chronic hepatitis C. Hepatol. Int..

[44] Gutiérrez-Rojas L., de la Gándara Martín J.J., García Buey L., Uriz Otano J.I., Mena Á., Roncero C. (2023). Patients with severe mental illness and hepatitis C virus infection benefit from new pangenotypic direct-acting antivirals: Results of a literature review. Gastroenterol. Hepatol..

[45] Margusino-Framiñán L., Bobadilla-Pérez E., Cid-Silva P., Rodríguez-Sotelo A., Yáñez-Rubal J.C., Mena-de-Cea Á., Suárez-López F., Prieto-Pérez A., Giménez-Arufe V., Delgado-Blanco M. (2020). Effectiveness and safety of direct-acting antivirals in hepatitis C infected patients with mental disorders: Results in real clinical practice. J. Med. Virol..

[46] Sundberg I., Lannergård A., Ramklint M., Cunningham J.L. (2018). Direct-acting antiviral treatment in real world patients with hepatitis C not associated with psychiatric side effects: A prospective observational study. BMC Psychiatry.

[47] Patel N., Nasiri M., Koroglu A., Bliss S., Davis M., McNutt L.A., Miller C. (2015). A Cross-Sectional Study Comparing the Frequency of Drug Interactions After Adding Simeprevir- or Sofosbuvir-Containing Therapy to Medication Profiles of Hepatitis C Monoinfected Patients. Infect. Dis. Ther..

[48] Shiner B., Watts B.V., Rozema L., Gradus J.L. (2025). Comparative Effectiveness of Direct-Acting Antiviral Treatment for Hepatitis C Virus Infection on Depressive Symptoms in Patients with Posttraumatic Stress Disorder. Clin. Ther..

[49] Tang L.S., Masur J., Sims Z., Nelson A., Osinusi A., Kohli A., Kattakuzhy S., Polis M., Kottilil S. (2016). Safe and effective sofosbuvir-based therapy in patients with mental health disease on hepatitis C virus treatment. World J. Hepatol..

[50] Mangia A., Milligan S., Khalili M., Fagiuoli S., Shafran S.D., Carrat F., Ouzan D., Papatheodoridis G., Ramji A., Borgia S.M. (2020). Global real-world evidence of sofosbuvir/velpatasvir as simple, effective HCV treatment: Analysis of 5552 patients from 12 cohorts. Liver Int..

[51] Brunetto M.R., Bonino F. (2025). The Natural History of Hepatitis C Virus Infection and Disease in the Era of Curative Therapy with Direct-Acting Antivirals. Viruses.

[52] Bird K., Socías M.E., Ti L. (2018). Integrating hepatitis C and addiction care for people who inject drugs in the era of direct-acting antiviral therapy. Int. J. Drug Policy.

[53] Grebely J., Tran L., Degenhardt L., Dowell-Day A., Santo T., Larney S., Hickman M., Vickerman P., French C., Butler K. (2021). Association Between Opioid Agonist Therapy and Testing, Treatment Uptake, and Treatment Outcomes for Hepatitis C Infection Among People Who Inject Drugs: A Systematic Review and Meta-Analysis. Clin. Infect. Dis..

[54] Jiang X., Song H.J., Wang W., Henry L., Childs-Kean L.M., Re V.L., Park H. (2021). The use of all-oral direct-acting antivirals in hepatitis C virus-infected patients with substance use disorders. J. Manag. Care Spec. Pharm..

[55] Martinello M., Naggie S., Rockstroh J.K., Matthews G.V. (2023). Direct-Acting Antiviral Therapy for Treatment of Acute and Recent Hepatitis C Virus Infection: A Narrative Review. Clin. Infect. Dis..

[56] Byrne C.J., Radley A., Inglis S.K., Beer L., Palmer N., Duc Pham M., Allardice K., Wang H., Robinson E., Hermansson M. (2022). Reaching people receiving opioid agonist therapy at community pharmacies with hepatitis C virus: An international randomised controlled trial. Aliment. Pharmacol. Ther..

[57] Fagiuoli S., Toniutto P., Coppola N., Ancona D.D., Andretta M., Bartolini F., Ferrante F., Lupi A., Palcic S., Rizzi F.V. (2023). Italian Real-World Analysis of the Impact of Polypharmacy and Aging on the Risk of Multiple Drug-Drug Interactions (DDIs) in HCV Patients Treated with Pangenotypic Direct-Acting Antivirals (pDAA). Ther. Clin. Risk Manag..

[58] Schmidbauer C., Schwarz M., Schütz A., Schubert R., Schwanke C., Gutic E., Pirker R., Lang T., Reiberger T., Haltmayer H. (2021). Directly observed therapy at opioid substitution facilities using sofosbuvir/velpatasvir results in excellent SVR12 rates in PWIDs at high risk for non-adherence to DAA therapy. PLoS ONE.

[59] Nava F.A., Mangia A., Riglietta M., Somaini L., Foschi F.G., Claar E., Maida I., Ucciferri C., Frigerio F., Hernandez C. (2023). Analysis of Patients’ Characteristics and Treatment Profile of People Who Use Drugs (PWUDs) with and without a Co-Diagnosis of Viral Hepatitis C: A Real-World Retrospective Italian Analysis. Ther. Clin. Risk Manag..

[60] EUDA European Union Drug Agency Statistical Bulletin 2025—Seizures of Drugs. https://www.euda.europa.eu/data/stats2025/szr_en.

[61] Piekarska A., Flisiak R., Pawłowska M., Jaroszewicz J., Gil L., Giebel S., Hus I., Lech-Marańda E., Tomasiewicz K. (2024). Recommendations of Polish HCV Expert Group and Polish Society of Haematologists and Transfusiologists on diagnosis and treatment of HCV-infected hemato-oncology patients. Acta Haematol. Pol..

[62] Mikulska M., van Bömmel F., Mouliade C., Indolfi G., Kefalakes H., von Lilienfeld-Toal M., Pischke S., Hermine O., Moradpour D., Wedemeyer H. (2025). Updated recommendations for the management of hepatitis B, C, and E virus infections in patients with haematological malignancies and those undergoing haematopoietic cell transplantation: Recommendations from the 9th European Conference on Infections in Leukaemia (ECIL-9). Lancet Haematol..

[63] Merli M., Carli G., Arcaini L., Visco C. (2016). Antiviral therapy of hepatitis C as curative treatment of indolent B-cell lymphoma. World J. Gastroenterol..

[64] Lockart I., Yeo M.G.H., Hajarizadeh B., Dore G.J., Danta M. (2022). HCC incidence after hepatitis C cure among patients with advanced fibrosis or cirrhosis: A meta-analysis. Hepatology.

[65] Celsa C., Stornello C., Giuffrida P., Giacchetto C.M., Grova M., Rancatore G., Pitrone C., Di Marco V., Cammà C., Cabibbo G. (2022). Direct-acting antiviral agents and risk of Hepatocellular carcinoma: Critical appraisal of the evidence. Ann. Hepatol..

[66] Cabibbo G., Celsa C., Calvaruso V., Petta S., Cacciola I., Cannavò M.R., Madonia S., Rossi M., Magro B., Rini F. (2019). Rete Sicilia Selezione Terapia—HCV (RESIST-HCV) and Italian Liver Cancer (ITA.LI.CA.) Group. Direct-acting antivirals after successful treatment of early hepatocellular carcinoma improve survival in HCV-cirrhotic patients. J. Hepatol..

[67] Rich N.E., Singal A.G. (2021). Direct-Acting Antiviral Therapy and Hepatocellular Carcinoma. Clin. Liver Dis..

[68] Giannini E.G., Pasta A., Plaz Torres M.C., Pieri G., Cabibbo G., Sangiovanni A., Piscaglia F., Campani C., Missale G., Vidili G. (2025). Absence of Viral Replication Is Associated with Improved Outcome in Anti-HCV-Positive Patients with Hepatocellular Carcinoma. Liver Int..

[69] Stella L., Cabibbo G., Celsa C., Ciccia R., Sparacino A., Piscaglia F., Tovoli F., Arleo A., Stefanini B., Iavarone M. (2025). Hepatitis C Eradication Improves Oncologic and Clinical Outcomes in Patients Treated with Atezolizumab Plus Bevacizumab. Liver Int..

[70] Italian Association for the Study of the Liver (AISF) (2019). AISF position paper on HCV in immunocompromised patients. Dig. Liver Dis..

[71] Mustafayev K., Torres H. (2022). Hepatitis B virus and hepatitis C virus reactivation in cancer patients receiving novel anticancer therapies. Clin. Microbiol. Infect..

[72] Persico M., Aglitti A., Caruso R., De Renzo A., Selleri C., Califano C., Abenavoli L., Federico A., Masarone M. (2018). Efficacy and safety of new direct antiviral agents in hepatitis C virus-infected patients with diffuse large B-cell non-Hodgkin’s lymphoma. Hepatology.

[73] American Association for the Study of Liver Diseases (2024). The Liver Meeting, 15–19 November 2024, San Diego, CA, USA.

[74] Torres H.A., Economides M.P., Angelidakis G., Hosry J., Kyvernitakis A., Mahale P., Jiang Y., Miller E., Blechacz B., Naing A. (2019). Sofosbuvir-Based Therapy in Hepatitis C Virus-Infected Cancer Patients: A Prospective Observational Study. Am. J. Gastroenterol..

[75] Punjala S.R., Logan A.J., Subramanian J., Von Stein L., Limkemann A., Al-Ebrahim M., Black S., Schenk A.D., Washburn W.K., Singh N. (2025). Outcomes of Liver Transplantation from Hepatitis C Virus-Positive DCD Donors and Its Utilization Among Centers in the United States. Transplantation.

[76] Kling C.E., Perkins J.D., Biggins S.W., Johnson C.K., Limaye A.P., Sibulesky L. (2019). Listing practices and graft utilization of hepatitis C-positive deceased donors in liver and kidney transplant. Surgery.

[77] Trapero-Marugán M., Little E.C., Berenguer M. (2018). Stretching the boundaries for liver transplant in the 21st century. Lancet Gastroenterol. Hepatol..

[78] Alqahtani S.A., Stepanova M., Al Shabeeb R., Eberly K.L., Ong J., Younossi Z.M. (2024). The impact of hepatitis B and C positive serologies on the outcomes of non-hepatic solid organ transplantation in the United States. J. Viral Hepat..

[79] Weinfurtner K., Reddy K.R. (2021). Hepatitis C viraemic organs in solid organ transplantation. J. Hepatol..

[80] Raasikh T., Jamali T., Flores A., Cotton R.T., Ramanathan V., Tan H.P., Hernaez R. (2021). Systematic review: Hepatitis C viraemic allografts to hepatitis C-negative recipients in solid organ transplantation. Aliment. Pharmacol. Ther..

[81] Brzdęk M., Zarębska-Michaluk D., Tronina O., Laurans Ł., Janczewska E., Dybowska D., Parfieniuk-Kowerda A., Tudrujek-Zdunek M., Białkowska-Warzecha J., Janocha-Litwin J. (2026). Treatment with Direct-Acting Antivirals in Patients with HCV Infection After Liver Transplantation. J. Clin. Med..

[82] Agarwal K., Castells L., Müllhaupt B., Rosenberg W.M.C., McNabb B., Arterburn S., Camus G., McNally J., Stamm L.M., Brainard D.M. (2018). Sofosbuvir/velpatasvir for 12 weeks in genotype 1-4 HCV-infected liver transplant recipients. J. Hepatol..

[83] Chen R., Li D., Zhang M., Yuan X. (2021). Sofosbuvir/Velpatasvir Prophylaxis for 12 weeks in Hepatitis C Virus (HCV)-Negative Recipients Receiving Kidney Transplantation from HCV-Positive Donors. Ann. Transplant..

[84] Rivosecchi R.M., Sanchez P.G., Sacha L.M., Horn E.T., Keebler M., Silveira F.P. (2025). A single center experience of concomitant administration of sofosbuvir/velpatasvir and amiodarone after heart and lung transplantation. JHLT Open.

[85] Mikulska M., Knelange N., Nicolini L.A., Tridello G., Santarone S., Di Bartolomeo P., de la Camara R., Cuéllar C., Velardi A., Perruccio K. (2022). Efficacy, safety and feasibility of treatment of chronic HCV infection with directly acting agents in hematopoietic stem cell transplant recipients—Study of infectious diseases working party of EBMT. J. Infect..

[86] AlKaabi H., AlSinani S., El-Kassas M., Alswat K.A., AlNaamani K.M. (2025). Hepatitis C Direct-Acting Antivirals in the Immunosuppressed Host: Mechanisms, Interactions, and Clinical Outcomes. Viruses.

[87] Makovich Z., Radosavljevic I., Chapyala S., Handley G., Pena L., Mok S., Friedman M. (2024). Rationale for Hepatitis C Virus Treatment During Hematopoietic Stem Cell Transplant in the Era of Novel Direct-Acting Antivirals. Dig. Dis. Sci..

[88] Cunningham H.E., Shea T.C., Grgic T., Lachiewicz A.M. (2019). Successful treatment of hepatitis C virus infection with direct-acting antivirals during hematopoietic cell transplant. Transpl. Infect. Dis..

[89] (2022). Kidney Disease: Improving Global Outcomes (KDIGO) Diabetes Work Group. *KDIGO* 2022 Clinical Practice Guideline for Diabetes Management in Chronic Kidney Disease. Kidney Int..

[90] Burgess S., Partovi N., Yoshida E.M., Erb S.R., Marquez Azalgara V., Hussaini T. (2015). Drug Interactions with Direct-Acting Antivirals for Hepatitis C: Implications for HIV and Transplant Patients. Ann. Pharmacother..

[91] Dugan E., Blach S., Biondi M., Cai Z., DePaola M., Estes C., Feld J., Gamkrelidze I., Kottilil S., Ma S. (2021). Global prevalence of hepatitis C virus in women of childbearing age in 2019: A modelling study. Lancet Gastroenterol. Hepatol..

[92] El-Shabrawi M.H.F., Kamal N.M., Mogahed E.A., Elhusseini M.A., Aljabri M.F. (2019). Perinatal transmission of hepatitis C virus: An update. Arch. Med. Sci..

[93] Schillie S., Wester C., Osborne M., Wesolowski L., Ryerson A.B. (2020). CDC Recommendations for Hepatitis C Screening Among Adults—United States, 2020. MMWR Recomm. Rep..

[94] Dobrowolska K., Zarębska-Michaluk D., Pawłowska M., Tudrujek-Zdunek M., Lorenc B., Berak H., Janczewska E., Mazur W., Janocha-Litwin J., Klapaczyński J. (2025). Sex-related differences in patients with chronic hepatitis C infection treated with direct-acting antiviral drugs. World J. Hepatol..

[95] Chappell C.A., Kiser J.J., Brooks K.M., Randolph R., Macio I.S., Meyn L.A., MaWhinney S., Rick A.M., Muniz G.B., Kwon K.M. (2025). Sofosbuvir/Velpatasvir Pharmacokinetics, Safety, and Efficacy in Pregnant People with Hepatitis C Virus. Clin. Infect. Dis..

[96] Giles M.L., Dunbar A., Krishnaswamy S., Sasadeusz J., Said J.M., Roon L., Bushman L.R., Brooks K.M. (2025). Pharmacokinetics of Sofosbuvir and Velpatasvir for Hepatitis C Treatment in Pregnancy. Biomedicines.

[97] German P., Moorehead L., Pang P., Vimal M., Mathias A. (2014). Lack of a clinically important pharmacokinetic interaction between sofosbuvir or ledipasvir and hormonal oral contraceptives norgestimate/ethinyl estradiol in HCV-uninfected female subjects. J. Clin. Pharmacol..

[98] Bastos F.I., Bastos L.S., Coutinho C., Toledo L., Mota J.C., Velasco-de-Castro C.A., Sperandei S., Brignol S., Travassos T.S., Dos Santos C.M. (2018). HIV, HCV, HBV, and syphilis among transgender women from Brazil: Assessing different methods to adjust infection rates of a hard-to-reach, sparse population. Medicine.

[99] Moradi G., Soheili M., Rashti R., Dehghanbanadaki H., Nouri E., Zakaryaei F., Amini E.E., Baiezeedi S., Ahmadi S., Moradi Y. (2022). The prevalence of hepatitis C and hepatitis B in lesbian, gay, bisexual and transgender populations: A systematic review and meta-analysis. Eur. J. Med. Res..

[100] Cocchetti C., Romani A., Mazzoli F., Ristori J., Lagi F., Meriggiola M.C., Motta G., Pierdominici M., Bartoloni A., Vignozzi L. (2022). Prevalence and Correlates of Sexually Transmitted Infections in Transgender People: An Italian Multicentric Cross-Sectional Study. J. Clin. Med..

[101] Sánchez-Toscano E., Collazo-Yáñez D., Domínguez-Riscart J., Larrán-Escandón L., Martín-Aspas A., Mateo-Gavira I., Aguilar-Diosdado M. (2025). Screening for sexually transmitted infections in the transgender population and interaction of gender-affirming hormonal treatment with antiretroviral therapy. Enfermedades Infecc. Microbiol. Clin. (Engl. Ed.).

[102] Cirrincione L.R., Senneker T., Scarsi K., Tseng A. (2020). Drug Interactions with Gender-Affirming Hormone Therapy: Focus on Antiretrovirals and Direct Acting Antivirals. Expert Opin. Drug Metab. Toxicol..

